# Automatic patient positioning and gating window settings in respiratory‐gated stereotactic body radiation therapy for pancreatic cancer using fluoroscopic imaging

**DOI:** 10.1002/acm2.12258

**Published:** 2018-01-27

**Authors:** Niclas Pettersson, Daniel Simpson, Todd Atwood, Jona Hattangadi‐Gluth, James Murphy, Laura Cerviño

**Affiliations:** ^1^ Department of Radiation Oncology University of California San Diego La Jolla CA USA

**Keywords:** automatic patient setup, fluoroscopy, hypofractionated radiation therapy, pancreatic cancer

## Abstract

Before treatment delivery of respiratory‐gated radiation therapy (RT) in patients with implanted fiducials, both the patient position and the gating window thresholds must be set. In linac‐based RT, this is currently done manually and setup accuracy will therefore be dependent on the skill of the user. In this study, we present an automatic method for finding the patient position and the gating window thresholds. Our method uses sequentially acquired anterior–posterior (AP) and lateral fluoroscopic imaging with simultaneous breathing amplitude monitoring and intends to reach 100% gating accuracy while keeping the duty cycle as high as possible. We retrospectively compared clinically used setups to the automatic setups by our method in five pancreatic cancer patients treated with hypofractionated RT. In 15 investigated fractions, the average (±standard deviation) differences between the clinical and automatic setups were −0.4 ± 0.8 mm, −1.0 ± 1.1 mm, and 1.8 ± 1.3 mm in the left–right (LR), the AP, and the superior–inferior (SI) direction, respectively. For the clinical setups, typical interfractional setup variations were 1–2 mm in the LR and AP directions, and 2–3 mm in the SI direction. Using the automatic method, the duty cycle could be improved in six fractions, in four fractions the duty cycle had to be lowered to improve gating accuracy, and in five fractions both duty cycle and gating accuracy could be improved. Our automatic method has the potential to increase accuracy and decrease user dependence of setup for patients with implanted fiducials treated with respiratory‐gated RT. After fluoroscopic image acquisition, the calculated patient shifts and gating window thresholds are calculated in 1–2 s. The method gives the user the possibility to evaluate the effect of different patient positions and gating window thresholds on gating accuracy and duty cycle. If deemed necessary, it can be used at any time during treatment delivery.

## 1 INTRODUCTION

Fiducial markers are commonly used in respiratory‐gated stereotactic body radiation therapy (SBRT) of pancreatic cancer^1^, ^2^, ^3^, ^4^ to assist in the visualization of the treatment area. SBRT of the pancreas needs a very accurate patient setup because small margins are used around the target to minimize dose and toxicity to surrounding organs at risk (OARs) such as the duodenum. Fiducials are used together with monitoring of patient breathing during patient setup and treatment.^5^ In gated treatments, proper setup of the patient requires that not only patient's position but also the gating window thresholds agree with the treatment plan. Different commercial treatment systems use various motion management methods for patient setup and/or intrafractional position monitoring.^6^, ^7^, ^8^, ^9^ However, current linear accelerators do not have the functionality to perform automatic setup of the patients and gating window based on fiducials, and rather rely on a manual patient setup.^10^ SBRT treatments, therefore, could benefit from the assistance of automatic, user‐independent methods.

Cone‐beam CT (CBCT) and fluoroscopic images are frequently used in image‐guided RT of pancreatic cancer to position the patient prior to treatment delivery. CBCT provides good soft‐tissue contrast, but is acquired throughout several breathing cycles, and is thus affected by the whole respiratory‐induced motion range. The resulting images, including fiducials, the tumor, and healthy organs, will therefore be blurred and difficult to use for patient positioning.^11^ Fluoroscopic images, frequently acquired after CBCT to verify or further refine setup, offer, on the other hand, high‐resolution, real‐time image information of the fiducials’ positions. A human observer (therapist, medical physicist, radiation oncologist) visually compares the acquired fluoroscopic images (the fiducials in real time moving throughout the respiratory cycle) with reference images from the treatment planning system, and decides how the patient should be positioned. The high temporal resolution and high contrast of fiducials on x‐ray images assist in making this decision. Due to the fiducials' high intensity in the fluoroscopic images, they can be automatically detected^12^, ^13^, ^14^, ^15^, ^16^ and have the potential to be automatically matched to the reference image.^10^

During respiratory‐gated RT, a breathing signal is typically acquired by externally measuring the anterior–posterior (AP) position of the chest or the abdomen. Assuming that the relationship between the internal position of the tumor and the external breathing signal does not change during the treatment fraction, the latter can then be used to identify when the tumor is at the correct position for treatment, that is, within the gating window.^5^ During setup, both the fiducials and the breathing signal need to be observed in order to set the patient position and the gating window thresholds. Due to a lack of both built‐in functionality and a scarcity of suggested methods that are applicable to most conventional linear accelerators, this process is currently done manually by the user, and the patient setup and corresponding treatment delivery accuracy are therefore user dependent.^10^

Recently, Wan et al. have developed a method to perform automatic setup (patient position and gating window) based on CBCT images.^11^ While CBCT images are routinely acquired during setup of SBRT patients, they take a considerable amount of time to acquire and would not be an ideal imaging technique to use in the middle of treatment if the position or internal–external tumor‐surrogate correlation needs to be verified. Fluoroscopic images, on the other hand, are quicker and simpler to acquire. In this study, we present a user‐independent automatic method of simultaneously finding an optimized patient position and gating window thresholds in patients with implanted fiducial markers for pancreatic treatments treated on a linear accelerator with a single kV imager. This is the first study to the best of our knowledge that presents such a method for conventional linear accelerators. The method is based on sequentially acquired fluoroscopic images in the lateral and AP directions, which are easily and quickly acquired when deemed necessary. We retrospectively compared clinically used setups to the automatic setups by our method in a group of pancreatic cancer patients.

## 2 METHODS

### 2.A Diagnostic imaging and fiducial contouring

We acquired data from five pancreatic cancer patients treated with SBRT at UC San Diego during 2016. Patients selected had two to four cylindrically shaped gold fiducials (diameter = 0.8 mm, length = 3 mm; MTNW887808, CIVCO Medical Solutions, Kalona, IA, USA) implanted in the tumor. This study was approved by the Institutional Review Board. Patients underwent free‐breathing CT scans (GE Lightspeed, GE Health Care, Pasadena, CA, USA) and the RPM system (Varian Medical Systems, Palo Alto, CA, USA) was used to monitor breathing motion during image acquisition. A 4DCT was created by using the phase information of the breathing signal to bin the images into ten phases in steps of 10%, where the 0% phase corresponds to end‐of‐inhale and the 50% phase to end‐of‐exhale. Since the treatment protocol uses end‐of‐exhale gating,^17^ the average intensity, the minimum intensity, and the maximum intensity projections (MIP) CT image sets built from the 30% to 70% phases (CT_3070av_, CT_3070min_ and CT_3070MIP_, respectively) were reconstructed and exported to the treatment planning system (TPS; Eclipse, Varian Medical Systems, Palo Alto, CA, USA). The spatial resolution of the images was 2.5 mm in the superior–inferior (SI) direction and 0.98–1.27 mm in the axial plane.

Using the CT_3070av_, CT_3070min_, and CT_3070MIP_ images as well as additional PET imaging, an internal target volume (ITV) was contoured in the TPS. The planning target volume (PTV) was created from the ITV using an isotropic 3‐mm margin. We contoured the fiducials on the CT_3070MIP_ images, and in some cases added an isotropic 1‐mm margin. The fiducial contours were then copied onto the CT_3070av_ to include them in the plan. AP and lateral digitally reconstructed radiographs (DRRs) containing the projected fiducial contours were calculated to assist in patient setup and were also exported for analysis.

### 2.B Clinical setup procedure

The following procedure was utilized to set up the patient at each fraction using amplitude‐based respiratory‐gated RT on a TrueBeam linear accelerator (Varian Medical Systems, Palo Alto, CA, USA) with the RPM system. The RPM box was placed and fixated on the patient, typically slightly below the sternum at the xiphoid process. Patients were instructed to breath freely and were not visually coached. The patient was initially positioned by orthogonal kV/kV imaging and then the setup was refined by matching the fiducials on CBCT images to their positions on the CT_3070av_. As the final step in patient setup, simultaneous fluoroscopic imaging and breathing signal monitoring was performed. When the breathing signal falls outside of the gating window, the fiducial contours from the DRRs images change color. The patient position and the gating window thresholds were then fine‐tuned to make sure that the fiducials were inside the fiducial contours at all times when the breathing signal was inside the gating window. This was done for AP and for lateral, patient right‐to‐left, fluoroscopic imaging.

When the patient was deemed to be accurately positioned, we acquired one AP and one lateral fluoroscopic image sequence, typically lasting 15–20 s each, for analysis purposes before treatment delivery was started. Since the TrueBeam is equipped with a single kV image detector, the AP and lateral fluoroscopic imaging sequences were acquired sequentially with a 90‐degree gantry rotation taking place between them. The fluoroscopic mages were acquired at a frame rate of 14.8 times per second, at 1500 mm source‐detector‐distance (SDD) and had a pixel size of 0.388 × 0.388 mm^2^. The source‐axis‐distance (SAD) was 1000 mm.

### 2.C Fiducial tracking

The fluoroscopic images and the RPM data were imported in Matlab (version 2014b, MathWorks, Natick, MA, USA) for analysis. In order to develop the automatic setup procedure, the position and motion of the fiducials during the breathing cycle and the gating window is needed. The method of template matching was used for fiducial tracking.^18^ For each fiducial, we manually created one rectangular template shape for lateral imaging and one rectangular template shape for AP imaging using the first lateral and the first AP image from the first fraction. Each template shape contained one fiducial with a surrounding margin of a few pixels where the center of the template corresponded to the center of the fiducial. For each following fraction, the center pixel of each fiducial was found in the first AP and lateral fluoroscopic images. To create the fraction‐specific fiducial templates, we then matched these fiducial centers to the center of the template shapes and extracted the corresponding pixels from the fluoroscopic image.

To automatically find the fiducial center in a fluoroscopic image, we used the fiducial center for the preceding image to create a search region ten pixels larger than the template in all directions. To find how much the fiducials had moved between images, the fiducial templates and the search regions were evaluated by the normalized cross‐correlation^19^ as implemented by *normxcorr2* in Matlab. The tracked positions were visually inspected in all sequences.

### 2.D Estimation of in‐room fiducial positions

By using the projected fiducial positions on the fluoroscopic images acquired at SDD, our method will calculate how much the patient needs to be shifted to reach the optimized position. However, to accurately convert the projected fiducial positions into patient shifts, we need to take the divergence of the x‐ray beam between the fiducials and the detector into account. When calculating the fiducial AP position in the in‐room coordinate system, AP_room_, from its projection on the lateral fluoroscopic image, we therefore need to know its in‐room LR position, LR_room_. The same applies for the LR_room_ position. Considering the imaging geometry where one sequence is laterally acquired and the other sequence is taken in the AP direction, the following equations

(1) $$LR_{\mathrm{room}}\left(f\right)=\frac{SAD-AP_{\mathrm{room}}\left(f\right)}{\mathrm{SDD}}\times LR_{detector,AP}\left(f\right)$$

(2) $$AP_{\mathrm{room}}\left(f\right)=\frac{SAD+LR_{\mathrm{room}}\left(f\right)}{\mathrm{SDD}}\times AP_{detector,LAT}\left(f\right)$$

relate the detector coordinates to the room coordinates. Here, LR_room_ is defined positive toward the left hand and AP_room_ defined positive in the anterior direction for a patient in a head‐first supine position. To estimate LR_room_ and AP_room_ for a fiducial, we took an approach similar to Cho et al. and approximated LR_room_ by initially assuming that AP_room_ = 0 mm and vice versa.^20^ Then, we iteratively used eqs. (1) and (2) to improve the estimates until the differences between two iterations both were below 0.1 mm. This approach is numerically well‐behaved and converges after two to three iterations.

Since we acquire the AP and lateral fluoroscopic image series sequentially and the fiducials are moving with respiration, we cannot pair up images to make this estimation for every fiducial position. Instead, we estimated one representative AP_room_ and one representative LR_room_ per fiducial using the mid‐range fiducial LR_detector_ and AP_detector_ positions.

### 2.E Proposed method for automatic patient positioning and gating window setting

The general idea behind the method is to compare the tracked fiducial positions on the fluoroscopic images with their expected positions inside their projected contours on the DRRs. The lateral and AP fluoroscopic image sequences and the corresponding breathing signals can be acquired in any order, but at least one complete breathing cycle must be acquired for each direction. Our proposed method to automatically calculate the patient position and the gating window thresholds comprises three steps.

- Step I: Superior–inferior patient shift

**Figure 1:**
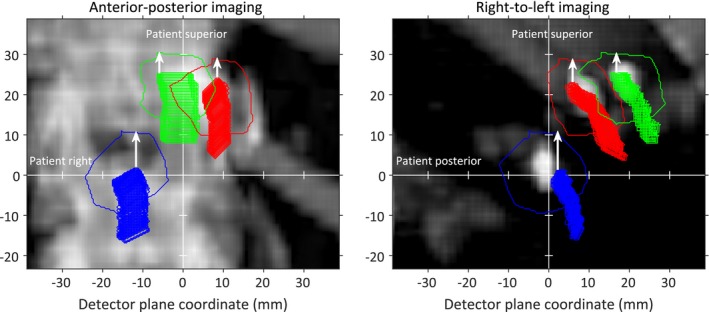
The tracked fiducial positions and projected fiducial contours on the DRRs (blue, red, and green). The differences between the overall most superior fiducial extents and the superior border of the projected contours (*dSI*_*detector*_) are shown as white arrows.

For each fiducial *f*, the difference between its overall most superior extent and the superior border of its projected contour on the DRR, *dSI*_*detector*_, was calculated for both AP and lateral imaging, as shown in Fig. 1, and converted to its in‐room differences according to

(3) $$dSI_{room,AP}\left(f\right)=\frac{SAD-AP_{\mathrm{room}}\left(f\right)}{\mathrm{SDD}}\times dSI_{detector,AP}\left(f\right)$$

(4) $$dSI_{room,LAT}\left(f\right)=\frac{SAD+LR_{\mathrm{room}}\left(f\right)}{\mathrm{SDD}}\times dSI_{detector,LAT}\left(f\right)$$

where the subscripts AP and LAT denote imaging direction. The optimized SI patient shift *dSI* is given by the smallest of *dSI*_*room*_. This shift is conservative in the sense that we do not allow any part of any fiducial to be more superiorly located than its projected contour.

- Step II: Lower and upper gating window thresholds

**Figure 2:**
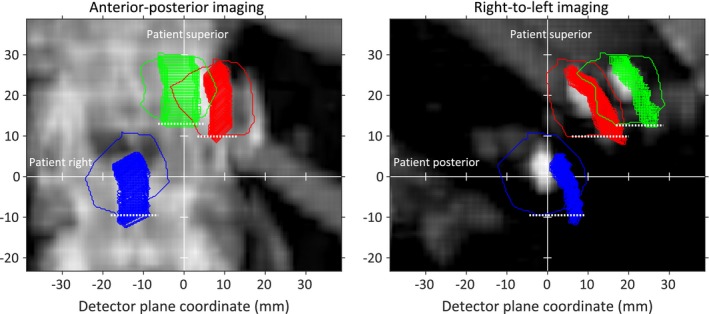
The tracked fiducial positions and projected fiducial contours on the DRRs (blue, red, and green) taking *dSI* into account. The inferior borders of the projected fiducial contours are shown using dashed white lines.

Taking *dSI* from Step I into account, we found the largest breathing amplitude for which no fiducial is more inferiorly positioned than the inferior border on its corresponding projected contour (Fig. 2). That breathing amplitude was set as the upper gating window threshold.

The lower gating window threshold is set as the overall smallest observed breathing amplitude. In a situation when the patient starts exhaling more deeply, this will prevent a beam on situation with fiducials located more superiorly than their projected contours.

- Step III: Left–right and anterior–posterior patient shifts

**Figure 3:**
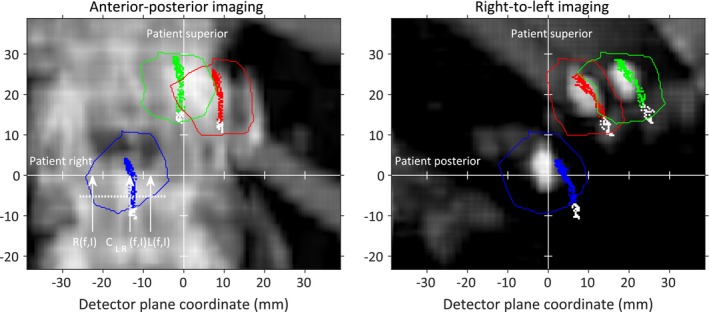
The center positions of the tracked fiducial and the projected fiducial contours on the DRRs (blue, red, and green) taking *dSI* into account. Fiducial positions outside the gating window are shown in white. The white arrows illustrate quantities in eq. (5) for one selected fiducial center position.

Using only the fiducial positions from the *N*_*1*_ AP images within the gating window and taking *dSI* from Step I into account, we calculated the LR patient shift *dLR* according to

(5) $$dLR=\frac{1}{M_{1}\times N_{1}}\times \sum_{f=1}^{M_{1}}\left(\frac{SAD-AP_{\mathrm{room}}\left(f\right)}{\mathrm{SDD}}\times \sum_{I=1}^{N_{1}}\frac{L\left(f,I\right)+R\left(f,I\right)}{2}-C_{\mathrm{LR}}\left(f,I\right)\right)$$

where *M*_*1*_ is the number of considered fiducials, *C*_*LR*_ *(f, I)* is the LR position for the center of fiducial *f* on image *I*,* L(f, I)* and *R(f, I)* are the left‐hand side and right‐hand side borders of the projected fiducial contours for the same SI position as *C*_*LR*_ *(f, I)*, respectively, as shown in Fig. 3. The *dLR* shift will center the considered fiducial positions within the projected contours in the LR direction.

The optimized AP patient position is found in an analogous way using the laterally acquired images. Using only fiducial positions from the *N*_*2*_ images within the gating window and taking *dSI* from Step I into account, we calculated the AP patient shift *dAP* according to

(6) $$dAP=\frac{1}{M_{2}\times N_{2}}\times \sum_{f=1}^{M_{2}}\left(\frac{SAD+LR_{\mathrm{room}}\left(f\right)}{\mathrm{SDD}}\times \sum_{I=1}^{N_{2}}\frac{A\left(f,I\right)+P\left(f,I\right)}{2}-C_{\mathrm{AP}}\left(f,I\right)\right)$$

where *M*_*2*_ is the number of considered fiducials, *C*_*AP*_ *(f, I)* is the AP position for the center of fiducial *f* on image *I*,* A(f, I)* and *P(f, I)* are the anterior and posterior borders of the projected fiducial contours for the same SI position as *CAP(f, I)*, respectively (Fig. 3). The shift *dAP* will center the considered fiducial positions within the projected contours in the AP direction.

The patient can now be shifted to the optimized position by moving the treatment table. Changing the vertical table position (*dAP*) will affect the position of the RPM marker box and the RPM signal and the gating window thresholds would need to be changed accordingly.

### 2.F Assessment of patient setup

**Figure 4:**
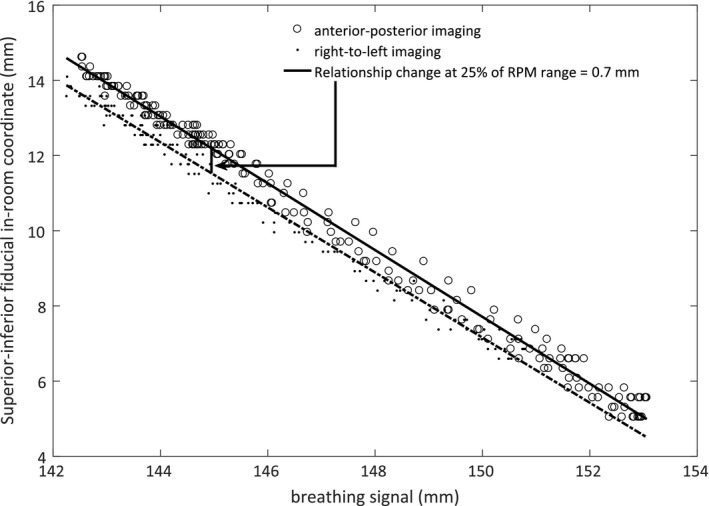
Example of quantification of the relationship change at 25% of the shared breathing signal range for one selected fiducial.

One of the assumptions behind using an external surrogate for respiratory gating is that the relationship between the external breathing signal and the tumor position is the same throughout the fraction. Since the SI fiducial positions are found at all imaging angles, the consistency of the RPM–fiducial relationship during setup can be evaluated. Taking the estimated LR_room_ and AP_room_ into account, we quantified the relationship change by the distance between the best linear fits at 25% of the shared breathing signal range as shown in Fig. 4. The 25% level was chosen since it in our case of 30%–70% gating would correspond to fiducial positions at or close to the center of its projected contour and thus gives equal weight to the superior and inferior borders. If several fiducials were considered, we defined the relationship change as the average change.

To assess gating accuracy, we classified a fiducial position as accurately gated if at least 75% of the fiducial was inside its projected contour.^11^, ^21^ The gating accuracy was then calculated as the percentage of accurate positions within the gating window averaged over all considered fiducials and both sequences. The duty cycle was defined as the percentage of time the breathing signal was within the gating window. We calculated the clinically used gating window thresholds from the recorded breathing amplitude and corresponding gating signal data (beam on/beam off).

We retrospectively compared the clinically used setups to the setups by our automatic method. Treatments were delivered in five fractions for all patients and five patients were available for analysis. In 15 of the 25 delivered fractions, we had imaging data for at least one breathing cycle for both sequences. The rest of the fractions were removed from the study. The number of implanted fiducials was two (*n* = 1), three (*n* = 3), or four (*n* = 1). For the patient with four implanted fiducials, two were not used for patient setup, and were therefore excluded from the analysis.

## 3 RESULTS

The average (±standard deviation, SD) relationship change between the breathing signal and the fiducial SI position for the lateral and AP projections was 0.6 ± 0.7 mm. In one case, the relationship change was larger than 2 mm (2.4 mm).

**Figure 5:**
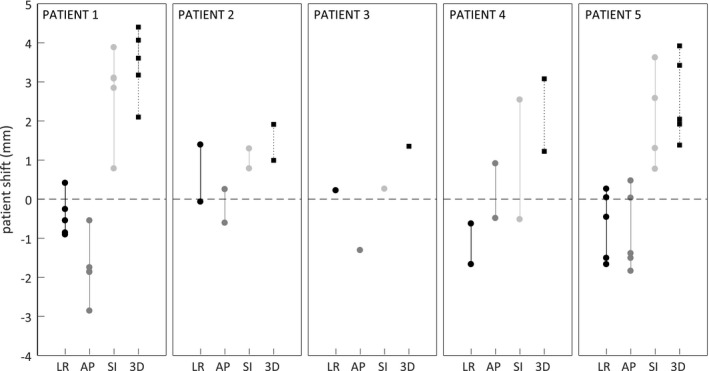
Calculated patient shifts in the left–right (LR, black), anterior–posterior (AP, gray), and superior–inferior (SI, light gray) directions for the automatic compared to the clinically used setups. The patient 3D shifts are shown using square black markers and dashed lines. Positive shifts are defined in the left, anterior, and superior directions.

All calculated patient shifts (i.e., the difference between the clinically used patient position and the automatically optimized patient position by our method) are summarized in Fig. 5. The overall mean (±SD) shifts were −0.4 ± 0.8 mm, −1.0 ± 1.1 mm, and 1.8 ± 1.3 mm in the LR, the AP, and the SI direction, respectively. There was a systematic 1.8 mm calculated patient shift (95% confidence interval: 1.1–2.5 mm) in the superior direction. The overall mean (±SD) for the calculated patient 3D shift was 2.6 ± 1.1 mm. The lengths of the vertical lines are the interfractional setup variations for the clinically used setups compared to the automatically optimized positions. Typical variations for the clinical setups were 1–2 mm in the LR and AP directions, and 2–3 mm in the SI direction.

**Figure 6:**
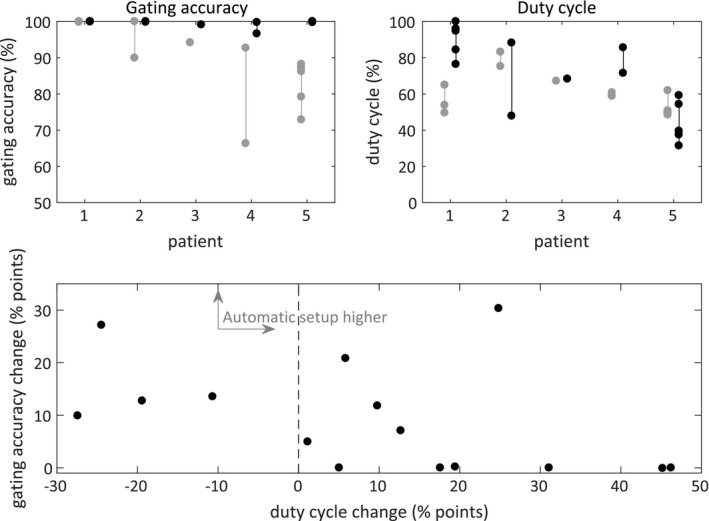
Upper left‐hand side panel: Gating accuracy comparison between the clinically used (gray) and automatic (black) setups. Upper right‐hand side panel: Duty cycle comparison between clinically used (gray) and automatic (black) setups. Lower panel: Change in gating accuracy vs change in duty cycle.

All gating accuracies and duty cycles for the clinically used and the automatic setups are shown in the upper panels of Fig. 6. The overall gating accuracy for the clinical setups was 90.4% ± 10.7% compared to 99.7% ± 0.9% for the automatic setups. In four fractions, the duty cycle had to be lowered to achieve this improvement; in six fractions with gating accuracy >95% for the clinical setups, accuracy could be maintained with increased duty cycles (27 ± 14 percentage points); and in five fractions, the gating accuracy and the duty cycle could be simultaneously improved.

## 4 DISCUSSION

In this study, we presented an automatic method for finding an optimized patient position and gating window thresholds for pancreatic cancer patients with fiducial markers treated with respiratory‐gated RT. The method employs sequentially acquired lateral and AP fluoroscopic images. Retrospective analysis of clinical patient setups showed that the automatic method has the potential to decrease the interfractional setup variation as well as to increase the gating accuracy and the duty cycle.

We chose to create a method where the primary goal was to achieve 100% gating accuracy while at the same time maximizing the duty cycle. As shown by the 29%–56% duty cycle variation for patient 5 in the upper right‐hand panel of Fig. 6, there is no guarantee that the breathing pattern during a setup matches the breathing pattern at 4DCT acquisition, and it is therefore of limited interest to aim for a specific duty cycle value. The method uses the same AP and lateral fluoroscopic imaging as our clinical protocol and once the images have been acquired, the time required to track the fiducials and calculate the optimized setup is about 1–2 s on a standard PC. If the resulting automatic setup is considered nonsatisfactory, the collected data can be used to evaluate different patient shifts and gating window settings without the need for additional imaging. In our clinical protocol, fluoroscopic imaging is the final setup step after initial kV/kV and CBCT positioning, and incorrect patient rotations can be expected to be small at this point. Therefore, the method does not consider rotations when finding the optimized patient position. Rotations are, however, possible to visually evaluate using the acquired images, and it would also be possible^20^ (but not implemented at this time) to evaluate any combination of couch rotations (yaw, pitch, and roll).

The proposed method is developed for end‐of‐exhale treatments, but can be modified for other types of gated treatment delivery. The collected data can also be used to check how much the relationship between the breathing signal and the fiducial SI position changed between the lateral and AP image acquisitions. If the user finds this change unacceptably large, some or all fluoroscopic images can be acquired again before proceeding with the final setup. Furthermore, the method can be applied at any time during treatment delivery if judged necessary due to for instance suspected patient movement or changed breathing pattern.

In our comparison of the clinically used and automatic setups, we considered the fiducials to have been accurately contoured in the treatment planning system. If the fiducials had been contoured differently, our patient shifts and gating accuracy assessments would differ from the ones shown in Figs. 5 and 6. The ranges of the LR, AP, and SI patient shifts in Fig. 5, however, represent the interfractional patient setup variations and they are independent of fiducial contour definition.

Our method employs two sequentially acquired 2D imaging projections. Since the fiducials are moving with respiration, this imaging geometry means that 3D triangulation of individual fiducial positions is not possible.^20^, ^22^, ^23^, ^24^, ^25^, ^26^ However, to be able to accurately calculate patient 3D shifts, x‐ray beam divergence still must be taken into account. This means that for AP imaging we need to know the LR fiducial position (and vice versa). We used eqs. (1) and (2) to make per‐fiducial position estimates at mid‐range of the motion and applied those estimates in eqs. (3), (4), (5), (6). Although a relatively simple approach, it has some advantages such as that no other imaging is required or for prior fiducial motion knowledge to be available while it at the same time produces sufficient accuracy. An error *dx* in the *AP*_*room*_ or *LR*_*room*_ estimate would affect *dSI*_*room*_ in eqs. (3) and (4) by *dy* = *dx* x *dSI*_*det*_/*SDD*. Among our collected clinical data (*n* = 10, including patients not included in this study), the maximum observed in‐room AP or LR motion range was 12 mm putting an realistic estimate of the worst‐case upper bound for *dx* at 12/2 = 6 mm. The introduced error *dy* for the calculated SI table shift will consequently be smaller than 0.3 mm for SI fiducial positions within 50 mm of the isocenter.

Wan et al. also created an automatic setup method for respiratory‐gated patients with implanted fiducials.^11^ In their method, they monitored the breathing amplitude during acquisition of the projection images used to create the CBCT for anatomical imaging. They tracked the fiducial locations on every projection image (*n* = 894) and optimized the patient position and gating window thresholds as a trade‐off between gating accuracy and duty cycle. They also found that their clinical setups could be improved upon by using an automatic method; in their case the average (±SD) patient 3D shift was 1.5 ± 0.8 mm. We have chosen fluoroscopic imaging for our study because it offers a faster way to check and recheck patient positioning and gating thresholds whenever needed before or during treatment.

Our work has been focused on the SBRT treatment of pancreatic cancer with implanted fiducials, where margins around the target are small to protect surrounding organs at risk such as the duodenum. In these cases, the accuracy of patient setup and gating windows is crucial for an accurate treatment. However, implanted fiducials are common in SBRT of the liver, and our methods are generalizable to such treatments.

Clinical implementation of the model will depend on the availability of a fiducial‐tracking algorithm. Several authors have investigated the performance of such algorithms.^12^, ^13^, ^14^, ^15^, ^16^, ^21^ Compared to more general tracking algorithms aiming to find the fiducials at any imaging angle, our method only requires them to be tracked in the lateral and AP projections. Although cylindrical fiducials were used to evaluate the method in this study; the method itself allows for fiducials of any size and shape as long as they can be accurately tracked. A clinical implementation would also ideally require little or no user intervention other than review and approval. Specifically, if template matching‐based fiducial tracking is used, the creation of the templates needs to be considered. To automatically create fiducial templates, Regmi et al. used pretreatment CT data while Wan et al. used the setup CBCT.^11^, ^14^ In the current software implementation of our method, fiducial template shapes are defined at the first fraction in a process that takes a few seconds per fiducial. These template shapes are then used for all subsequent fractions. Although we did not observe problems with the fiducial placement or tracking in this study, for the clinical implementation, we envision that implanted fiducials should be placed in different SI locations to ensure proper visibility of all of them in the fluoroscopic images, and that the radiation oncology team would always verify patient setup prior to treatment, ensuring the correct functioning of the algorithm, including cases of fiducial migration.

## 5 CONCLUSION

In conclusion, our proposed method uses sequentially acquired fluoroscopic images to automatically calculate patient position and gating window thresholds after an initial CBCT‐based alignment. Additionally, it provides a flexibility to investigate how different patient positions and gating window settings would affect gating accuracy and duty cycle and it can, if deemed necessary, be used at any time during treatment delivery. Setup of patients treated with respiratory‐gated RT is currently user dependent as well as time consuming. Having access to an automatic method to assist in setting the patient position and gating window thresholds could increase treatment delivery accuracy.

## CONFLICTS OF INTEREST

This study was funded by a research grant from Varian Medical Systems held by Dr Cerviño. Dr. Hattangadi‐Gluth has a research grant from Varian Medical Systems, unrelated to the current study.
